# Endoscopic ultrasonography-guided hepaticoduodenostomy using a novel self-expandable metallic stent with an ultra-tapered tip and a slim-delivery system

**DOI:** 10.1055/a-2590-8508

**Published:** 2025-05-19

**Authors:** Haruo Miwa, Yugo Ishino, Kazuki Endo, Ritsuko Oishi, Yuichi Suzuki, Yusuke Suwa, Shin Maeda

**Affiliations:** 1 26437 Gastroenterological Center, Yokohama City University Medical Center, Yokohama, Kanagawa, Japan; 2 26437 Department of Surgery, Gastroenterological Center, Yokohama City University Medical Center, Yokohama, Japan; 3 Department of Gastroenterology, Yokohama City University Graduate School of Medicine, Yokohama, Japan

Endoscopic ultrasonography-guided hepaticoduodenostomy (EUS-HDS) is an effective treatment for biliary obstruction of the posterior branch; however, inserting the stent delivery system can be challenging in cases with an acute angle to the bile duct 1 2 3 4 5 . A novel partially covered self-expandable metallic stent (Niti-S EUS-BD system End Bare Single Flare; Taewoong Medical Co., Ltd.) with an ultra-tapered tip specifically designed for a 0.025-inch guidewire and 7-Fr slim delivery system facilitates transluminal stent insertion. Herein, we report a challenging case of EUS-HDS using the novel 7-Fr Niti-S stent ( Video 1 ).

Endoscopic ultrasonography-guided hepaticoduodenostomy was successfully performed using a novel self-expandable metallic stent with an ultra-tapered tip and a 7-Fr slim delivery.Video 1

A 61-year-old woman with huge liver metastases of colon cancer was admitted due to hilar biliary obstruction. The intrahepatic bile duct was separately obstructed as Bismuth type IV. Initially, endoscopic ultrasonography-guided hepaticogastrostomy was performed; however, jaundice did not improve sufficiently ( Fig. 1 ). Due to the presence of ascites around the right liver lobe, EUS-HDS was attempted as an alternative to percutaneous drainage ( Fig. 2 ). The right posterior branch was punctured using a 19-gauge needle. High resistance was felt during puncture because the Glisson sheath was penetrated ( Fig. 3 ). After the insertion of a 0.025-inch guidewire (VisiGlide 2; Olympus medical systems), an ultra-tapered catheter could not pass through the bile duct wall. Although a 7-Fr mechanical dilator (ES dilator soft type; Zeon Medical) was inserted into the posterior branch, the catheter still failed to advance. Subsequently, the novel 7-Fr Niti-S stent was attempted to insert into the bile duct. The ultra-tapered tip of the slim-delivery system easily advanced across the bile duct wall, and the 8-mm and 10-cm stent was successfully placed. Cholangiography confirmed no bile leakage into the abdominal cavity ( Fig. 4 ).

**Fig. 1:**
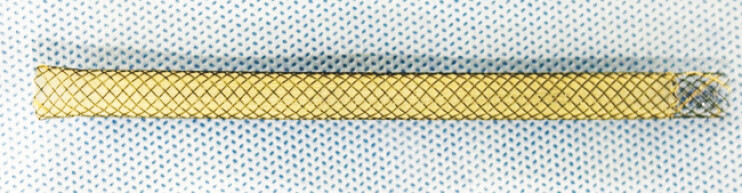
A novel 7-Fr Niti-S EUS-BD system End Bare Single Flare.

**Fig. 2:**
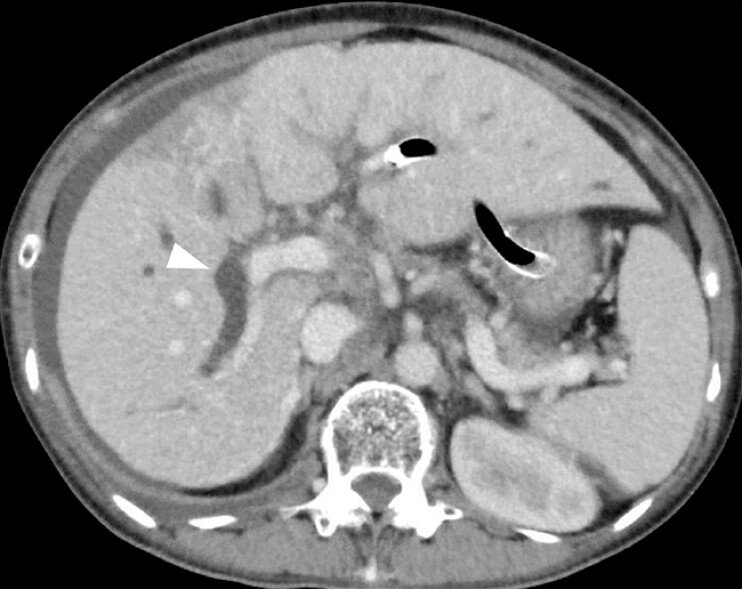
A computed tomography image after endoscopic ultrasonography-guided hepaticogastrostomy shows ascites and dilated posterior branches (arrowhead).

**Fig. 3:**
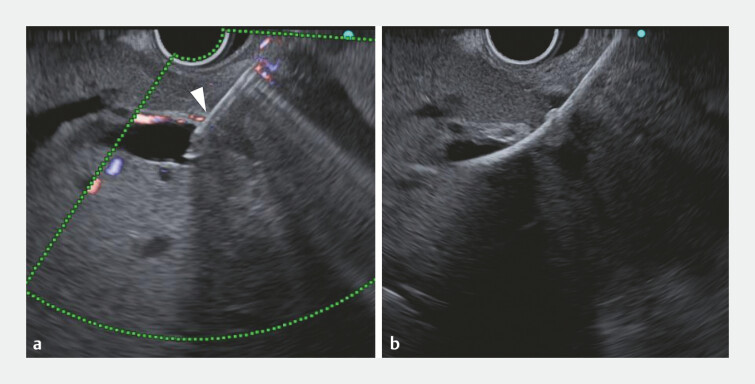
Ultrasound images during the procedure. **a** A 19-gauge needle penetrates the Glisson sheath around the bile duct (arrowhead). **b** A 7-Fr slim-delivery system of the novel Niti-S stent passes through the bile duct wall.

**Fig. 4:**
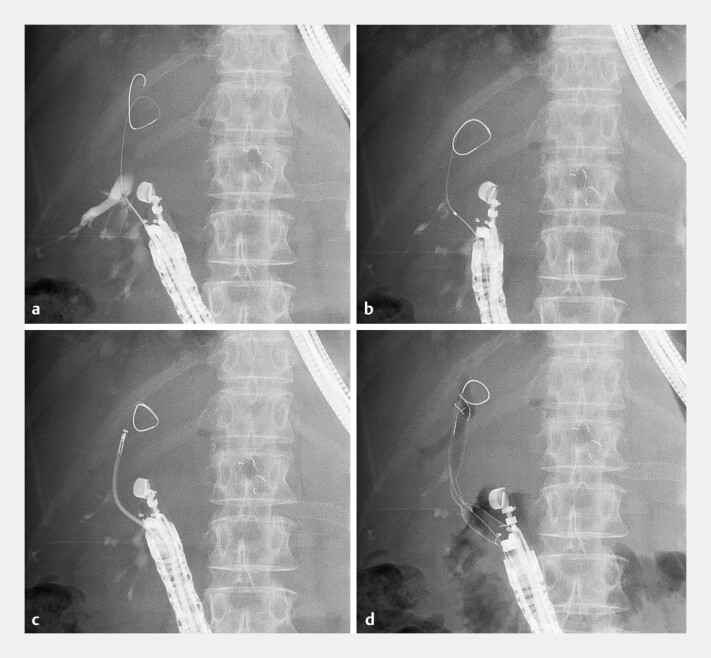
Fluoroscopic images during the procedure. **a** The posterior branch is punctured, and a guidewire is inserted into the bile duct. **b** An ultra-tapered catheter fails to advance after dilation. **c** The novel 7-Fr Niti-S stent can be inserted into the bile duct. **d** The stent is successfully deployed to the duodenum.

To the best of our knowledge, this is the first report of successful EUS-HDS using the novel 7-Fr Niti-S stent with an ultra-tapered tip and a slim-delivery system.

Endoscopy_UCTN_Code_TTT_1AS_2AD

## Notes

Endoscopy E-Videos https://eref.thieme.de/e-videos
*E-Videos* is an open access online section of the journal *Endoscopy* , reporting on interesting cases and new techniques in gastroenterological endoscopy. All papers include a high-quality video and are published with a Creative Commons CC-BY license. Endoscopy E-Videos qualify for HINARI discounts and waivers and eligibility is automatically checked during the submission process. We grant 100% waivers to articles whose corresponding authors are based in Group A countries and 50% waivers to those who are based in Group B countries as classified by Research4Life (see: https://www.research4life.org/access/eligibility/ ). This section has its own submission website at https://mc.manuscriptcentral.com/e-videos .
